# Colloidal Stability and Magnetic Field-Induced Ordering of Magnetorheological Fluids Studied with a Quartz Crystal Microbalance

**DOI:** 10.3390/s151229808

**Published:** 2015-12-04

**Authors:** Jaime Rodriguez-López, Pedro Castro, Juan de Vicente, Diethelm Johannsmann, Luis Elvira, Jose R. Morillas, Francisco Montero de Espinosa

**Affiliations:** 1 Institute of Physical and Information Technologies, CSIC, C/Serrano, 144, Madrid 28006, Spain; dr.jaimerl@gmail.com (J.R.-L.); luis.elvira@csic.es (L.E.); francisco.montero@csic.es (F.M.E.); 2 Department of Applied Physics, Faculty of Sciences, University of Granada, c/Fuentenueva s/n, Granada 18071, Spain; jvicente@ugr.es (J.V.); jrmorillasmedina@correo.ugr.es (J.R.M.); 3 Institute of Physical Chemistry, Clausthal University of Technology, Arnold-Sommerfeld-Str. 4, Clausthal Zellerfeld D-38678, Germany; johannsmann@pc.tu-clausthal.de

**Keywords:** TSM resonator, magnetorheological fluid, QCM, positive resonant frequency shift, micron-sized magnetic particles, sedimentation, magnetic field intensity

## Abstract

This work proposes the use of quartz crystal microbalances (QCMs) as a method to analyze and characterize magnetorheological (MR) fluids. QCM devices are sensitive to changes in mass, surface interactions, and viscoelastic properties of the medium contacting its surface. These features make the QCM suitable to study MR fluids and their response to variable environmental conditions. MR fluids change their structure and viscoelastic properties under the action of an external magnetic field, this change being determined by the particle volume fraction, the magnetic field strength, and the presence of thixotropic agents among other factors. In this work, the measurement of the resonance parameters (resonance frequency and dissipation factor) of a QCM are used to analyze the behavior of MR fluids in static conditions (that is, in the absence of external mechanical stresses). The influence of sedimentation under gravity and the application of magnetic fields on the shifts of resonance frequency and dissipation factor were measured and discussed in the frame of the coupled resonance produced by particles touching the QCM surface. Furthermore, the MR-fluid/QCM system has a great potential for the study of high-frequency contact mechanics because the translational and rotational stiffness of the link between the surface and the particles can be tuned by the magnetic field.

## 1. Introduction

Thickness shear mode (TSM) resonators (the QCM being the most widely employed type) consist of thin, AT-cut piezoelectric crystals covered with electrodes on both sides. When subjected to an alternating voltage, the plate vibrates, where the cut is chosen such that the mode of vibration is of the thickness-shear type. Excitation here occurs with broad-band RF pulses, which excite many different acoustic resonances at the same time. Given the high quality factor, the resonance frequencies can be easily determined with excellent precision. Since the crystal vibrates in the thickness-shear mode, it emits shear acoustic waves, which rapidly decay and turn the QCM into a surface-specific device [1]. The QCM is only sensitive to objects inside a spatial range limited by the penetration depth of the shear wave, δ = (2η/(ρω))^1/2^, where η and ρ are the viscosity and the density of the fluid, respectively, and ω is the angular frequency. The depth of penetration in water at MHz frequencies is of the order of 100 nm. Importantly, the resonance parameters of a TSM resonator change when the resonator surface is brought into contact with a sample. When the sample consists of a rigid film (<100 nm), a thin viscoelastic layer (<1 µm, depending on the softness), or a fluid, the resonance frequency decreases because the sample increases the effective mass of the resonator [2]. In other cases, the acoustic impedance seen by the resonator is reactive. Under these conditions, the resonance frequency increases. One such case is the coupled resonance [3].

TSMs devices were initially developed to monitor vapor deposition processes. These tools are very sensitive to changes in thickness (nm) or in mass (ng/cm^2^), which motivated the term “quartz crystal microbalance (QCM)”. While early applications were restricted to air or vacuum [4], Nomura showed in 1982 [5] that loading with a fluid is also possible. Since the system was not overdamped, one could still determine a resonance frequency. Additionally, the resonance bandwidth was shown to contain valuable information. The resonance bandwidth is often converted to an inverse Q-factor, *Q*^−1^, also called “dissipation factor”, *D*, or “dissipation”, for short. In Newtonian liquids, the dissipation factor is proportional to (ρη)^1/2^. These insights triggered the widespread application of the QCM in liquid environments [1]. Of particular importance has been the characterization of chemical and biological samples using these devices. The QCM is now used as a microgravimetric device or as a detector for adsorption to functionalized surfaces at many places [6,7,8,9,10].

As research progressed, it turned out that TSM resonators are not only sensitive to mass or thickness changes, but also to changes in the viscoelastic properties of the layer system [11]. Even more relevant to the topic of this work is the fact that the device is also sensitive to changes in the rigidity of contacts between particles and the resonator surface [12,13]. The ability to probe contact stiffness is critical to the use of such sensors for the characterization of MR fluids [1]. The sensitivity of the TSM devices provides new insights into these attractive and complex materials, which have not been previously studied with this technique.

Conventional magnetorheological (MR) fluids are suspensions of micron-sized magnetic particles in a viscous fluid. Their viscosity and viscoelasticity reversibly change when they are exposed to external magnetic fields [14,15]. With no magnetic field applied, the particles are dispersed randomly within the carrier fluid. However, if not properly stabilized, the particles settle. All the commercial formulations include stabilizers to prevent sedimentation issues. When a magnetic field is turned on, the particles become magnetized and interact with each other, leading to the formation of aggregates which are elongated along the direction of the field. This induces a certain amount of order and rigidity in the bulk which, in consequence, also strengthens the interactions between the particles and the surface of the container [16,17,18].

MR fluids are mostly employed as mechanical devices, building on the fact that the magnetic field changes the fluid rheological properties. For example, MR fluids are used as tunable clutches, breaks, or dampers in the automotive industry or even for sealing or polishing applications [19,20,21]. Many different factors influence the performance of MR fluids, including the particle volume fraction, the viscosity of the carrier fluid, and the properties of the particles [15,18]. Still, the principle driver for the control of the flow properties is the magnetic field. The higher the field strength, the stronger are the interactions between particles and the more rigid is the system.

In this work, TSM resonators have been used for the first time to characterize MR fluids in static conditions. Moreover, real-time monitoring of MR fluids by means of a TSM resonator is proposed as an effective tool to evaluate the quality of the system with regard to the efficiency of magnetic switching. Since TSM resonators are surface-specific, they are convenient tools to explore the kinetic stabilization and the rearrangement of particles once a magnetic field is applied. Sedimentation under gravity and the switching efficiency of the MR fluid are probed by incorporating a quartz resonator into to a torsional rheometer with parallel-plate geometry. Although TSM resonators are only sensitive to a thin interfacial region, the behavior of the bulk may still be inferred from the conditions close to the surface.

## 2. Background on High-Frequency Contact Mechanics

Conventional MR fluids are composed of heavy, micron-sized carbonyl iron spheres suspended in a carrier liquid. When in contact with the surface of the QCM sensor the dependence of the latter’s resonance frequency and the dissipation factor on the state of sedimentation and the magnetic field strength can be understood in the frame of the coupled resonance [3]. We briefly elaborate on the background.

Following the small-load approximation, the frequency shift, Δ*f*, and the shift of the half bandwidth, Δ*Γ*, obey the relation [22]:

(1) $$\Delta f+i\Delta \Gamma =\frac{if_{0}}{\pi Z_{q}}\frac{{\langle \sigma_{0}\rangle }_{area}}{i\omega u_{0}}=\frac{{\langle \sigma_{0}\rangle }_{area}}{2n\pi^{2}Z_{q}u_{0}}$$

The parameter *Г* is related to the dissipation factor, *D*, by *D* = *Г*/(2*f*). *f*_0_ is the fundamental frequency, *Z_q_* = 8.8 × 10^6^ kg · m^−2^s^−1^ is the shear-wave-specific acoustic impedance of AT-cut quartz, and 〈σ_0_〉*_area_* is the area-averaged complex amplitude of the tangential stress at the resonator surface. *n* is the overtone order and *u*_0_ is the amplitude of oscillation. The ratio of stress and velocity (where the latter is equal to i ω*u*_0_) is the complex load impedance, *Z_L_*.

For a thin rigid film (thinner than about 1 µm, depending on the film’s shear modulus), the stress is mostly of inertial origin (σ = iω*m* with *m* the mass per unit area) and the frequency shift is negative. There is an opposing limit, where the resonator surface is in contact with a finite number of discrete external objects. In this so-called elastic limit, these objects are so heavy that inertia clamps them in space. They neither rotate nor do they undergo translation. The size of the contact between the resonator and the external object (often a sphere) in this model is assumed to be much smaller than the wavelength of sound. If this is the case, the contact can be depicted as a spring [3]. The spring connects regions in the resonator and the sphere, which are hardly deformed at all. They either oscillate laterally at the resonator’s amplitude of motion (in the case of the substrate) or are at rest (in the case of the sphere). In other words, the deformation is limited to a range close to the point of contact; it scales as the inverse of the distance to the point of contact and there is a well-defined far-field limit, where the deformation induced by the contact is small. Again, if the sphere is heavy enough, the far-field limit inside the sphere is in the state of rest because the sphere is too heavy to follow the MHz motion of the crystal.

Given that the contact can be represented as a spring, Equation (1) can be expressed as,

(2) $$\Delta f+i\Delta \Gamma =\frac{if_{0}}{\pi Z_{q}}\frac{N}{A}\frac{\kappa }{i\omega }$$

The frequency shift is proportional to the stiffness of the link and inversely proportional to the overtone order. The parameter κ is the complex spring constant. *N*/*A* is the number of spheres per unit area. If the link dissipates energy, the spring constant turns complex (κ = κ′ + iκ′′ with κ′′ the dissipative part). A nonzero κ′′ gives rise to an increase in bandwidth in addition to an increase in frequency.

**Figure 1:**
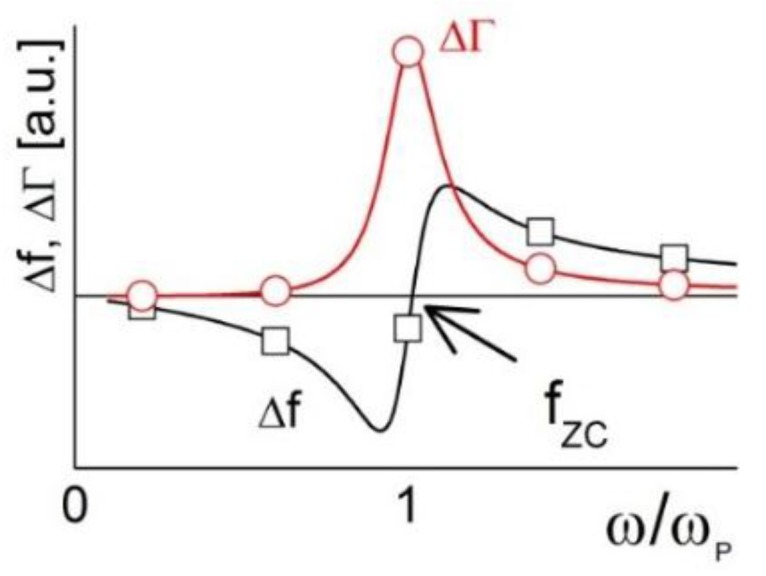
Shifts of frequency and bandwidth around a coupled resonance (23–25). *f_ZC_* is the frequency of zero crossing, which is similar to the particle resonance frequency.

The matter becomes more complicated if the external objects (the magnetic spheres, in this case) are not heavy enough to be clamped in space by inertia, but are still too heavy to rigidly follow the motion of the resonator surface. As will be explained later, this situation is encountered when the sample is not kinetically stable and particles sediment under gravity. It is characterized by what is called the “coupled resonance”. For the mathematical details, we refer the reader to the literature [3,12,23]. As for particles with a size in the range of a few micrometers (which are too heavy to rigidly follow the resonator’s motion and not heavy enough to be clamped in space), the particle together with the contact forms a resonator of its own. Its resonance frequency is called “particle resonance frequency”, ω*_P_*. If ω*_P_* is much larger than the resonance frequency of the QCM, inertial effects dominate [3] and Δ*f* is negative. If ω*_P_* is much smaller than the QCM-frequency, the elastic limit holds (see above). If ω*_P_* is comparable to the QCM frequency, one finds a crossover between negative and positive Δ*f* at an overtone order comparable in frequency to the particle resonance frequency ([24,25,26], see also Figure 1).

In principle, this picture can be turned into a quantitative model. The problem with the quantitative application of the model to MR fluids is that the numerous contacts vary in their properties. There is a distribution in mass, in the elastic contact stiffness, and in the dissipative part of the contact stiffness. Further, there is the coupled liquid as well as the possibility of hydrodynamic interactions between neighboring particles. Given these uncertainties, the results obtained in this work are discussed in the framework of the coupled resonance, but the discussion is kept at a qualitative level.

## 3. Experimental Section

### 3.1. Experimental Set-Up

**Figure 2:**
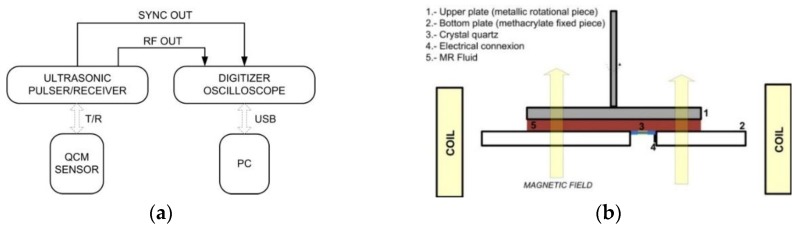
(**a**) Scheme of the experimental set up; and (**b**) measurement cell adapted to the rheometer (not to scale).

The TSM measuring technique is similar to the one described by Resa *et al.* [27], where a broadband spike (impulse) excitation method was used to interrogate the resonance parameters. The experimental setup is depicted in Figure 2 and consists of the following parts:

− A QCM cell containing an AT-cut quartz crystal provided by CH Instruments Inc. (Austin, TX, USA, 7.995 MHz fundamental frequency, 13.7 mm blank diameter, 5.1 mm electrode diameter, polished surface finish, 100 Å Ti and 1000 Å Au electrode material, keyhole electrode pattern).
− 200 MHz (−3 dB) Panametric ultrasonic pulser/receiver, model 5900 PR (P/R mode, 2 kHz PRF, 1 μJ energy, 50 Ω damping, 1 MHz HP filer, 200 MHz LP filter, 0 dB attenuators, 40 dB gain, 0˚ RF output phase).
− 500 MHz, 4 GSa/s, DS4054 Rigol, digitizing oscilloscope (150,000 acquisition points).
− Personal computer connected to the oscilloscope via USB interface.

The QCM cell was incorporated into a rotational shear rheometer as described in [28]. Briefly, the rheometer (Anton Paar MCR 501, Graz, Austria) consists in two parallel plates: the stationary methacrylate bottom plate and a metallic upper plate that can be rotated if necessary. The acoustic measurements were all undertaken under quiescent conditions. Measurements under applied stress are left for future work. The use of a rheometer permits a careful control of temperature, suspension thickness, magnetic field, and sample preconditioning by shear flow. Experiments were run under isothermal conditions using a Julabo refrigerated/heating circulator bath set at 25.00 ± 0.01 °C and a P-PTD 200 Peltier system. The distance between the two disks of the rheometer, which defines the sample thickness, was set at 0.6 mm. The magnetic field was generated with a custom-made coil, which generates a uniform magnetic field with strength of up to 15 kA/m.

The AT-cut quartz crystal was placed into a cylindrical hole cut into the bottom plate. The sensor was displaced from the center by 14 mm (Figure 2b). The electrodes of the resonator were connected to an RF coaxial cable using silver conductive glue. The electrode in contact with the sample was grounded, which avoids artifacts connected to piezoelectric stiffening [29]. The active surface area of the quartz was free of electrical and mechanical contacts to ensure the resonance was not significantly altered. The edge of the sensor was sealed to the methacrylate bottom plate using an epoxy resin. The QCM surface was aligned with the surface of the bottom plate to allow for a smooth flow of material past the resonator in the pre-shear stage.

The virtual programming environment LabVIEW was used to record the output signals; signal processing was performed with MATLAB. The time lapse between signal acquisition runs was 3 s.

### 3.2. Signal Processing

**Figure 3:**
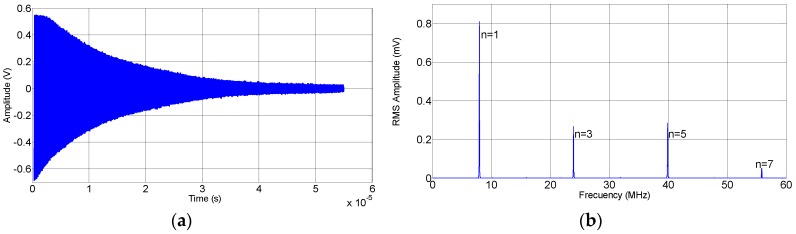
(**a**) Time trace of the signal on the oscilloscope; and (**b**) frequency spectrum derived from the time trace by Fourier transformation.

Figure 3 shows the waveform of the signal displayed on the oscilloscope (a) and its frequency spectrum (b). To determine the resonant frequencies a temporal interval (from 95% to 25% of signal) was selected. The signals were multiplied by a Hanning window that was zero-valued outside of the selected interval. The resonant frequencies were calculated from the maximum values of the zero-padded Fast Fourier Transform (FFT). The determination of these quantities was performed by using a cubic spline data interpolation (with steps of 10 Hz multiplied by *p*^1/2^, with *p =* (*n* + 1)/2, where *n* is the overtone order), in an interval centered at the resonance frequency with a variable span depending on the harmonic (2 MHz multiplied by *p*^1/2^).

The dissipation factor, *D* = *Q*^−1^ was calculated from the bandwidth, *B_W_*, as *D* = *B_W_*/*f*_0_. The bandwidth was determined from the decay rate of the time-domain signal. Supposing a monochromatic sinusoidal signal, *u*(*t*), of maximum amplitude, *u*_0_, with an exponentially decaying envelope:

(3) $$u(\text{t})=u_{0}e^{-\frac{t}{\tau }}\sin(2\pi f_{0}t+\phi )$$

the half bandwidth, Γ, is inversely proportional to the decay time constant, *τ*:

(4) $$\Gamma =\frac{B_{w}}{2}=\frac{1}{2\pi \tau }$$

**Figure 4:**
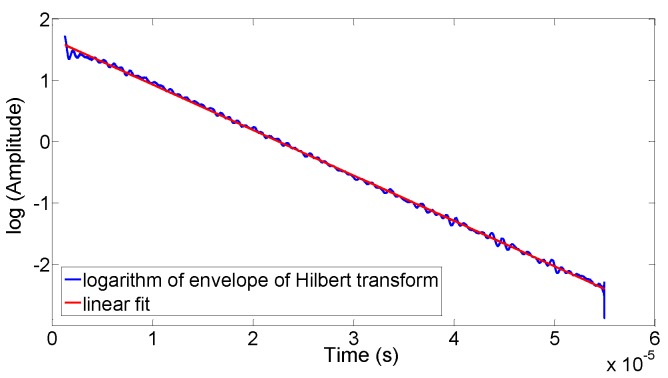
A fit of an exponential function to the envelopes of a monochromatic signal (obtained by a Hilbert transform). The decay constant is obtained from the fit.

After removing the DC offset, the output signals of each harmonic were separated applying a filter with 0.01 dB of peak-to-peak ripple and a minimum stopband attenuation of 40 dB. Subsequently, the spectral data were converted back to the time domain via the inverse FFT (via Hilbert transform). The envelopes of the ‘monochromatic’ signals were obtained from the amplitude of the resulting complex time signal. Since these envelopes were not subject to a pure exponential decay, only an intermediate range of the curves were fitted to an exponential function (the interval goes from 95% to 5% of signal). Figure 4 shows an example of a linear fit to the logarithm of the signal previously mentioned.

### 3.3. MR Fluid Characteristics

Two types of magnetorheological fluids with very different characteristics were used to study the effect of sedimentation; one non-commercial unstable suspension with a low particle concentration and no additives, and another highly-stable commercial suspension with a high particle loading and proprietary additives:

− The sample “OM MRF” is the homemade suspension. It consists of carbonyl iron microparticles (OM grade from BASF SE, mean diameter 5 μm) dispersed in a highly-viscous silicone oil (487 mPa·s, Sigma-Aldrich, St, Louis, MO, USA). The particle content was 5 vol%. This formulation does not include additives. The density of the suspensions at 25 °C was 1126 ± 10 kg/m^3^. The viscosity of the OM MRF measured with the rotational shear rheometer at a temperature of 25 °C and a shear rate of 100 s^−1^ was 515 ± 15 mPa·s.
− The sample “Commercial MRF” (reference MRF-132 supplied by Lord Corporation, Carrey, NC, USA) consists of iron particles with a diameter between 5 to 10 μm suspended in a carrier fluid. The particle volume fraction is 32 vol%. In this case, (proprietary) thixotropic additives prevent short-term sedimentation. According to the manufacturer, the density of the suspension at 25 °C is 3050 ± 100 kg/m^3^. The viscosity of the commercial MRF measured with the rotational shear rheometer at a temperature of 25 °C and a shear rate of 100 s^−1^ was 300 ± 50 mPa·s.

More detailed information on the MR fluids used in this work can be found in Table 1.

**Table 1:** Properties of the MR fluids used: OM MRF and Commercial MRF.

MR Fluids	Density kg/m^3^	Viscosity mPas	Vol. Fraction	Thixotropic Additives	Carrier Liquid	Particles	
**OM_MR**	^b^ 1126 ± 10	^b^ 515 ± 15	5%	NO	Silicone oil	Grade	OM
						Coating	None
						Diameter	5 µm
						Bulk composition	Fe(>97.8%), C(0.7%–0.9%), N(0.6%–0.9%), O(0.2%–0.4%)
**Commercial MRF**	^a^ 3050 ± 100	^b^ 300 ± 50	32%	YES	Hydrocarbon	Grade	Unknown
						Coating	YES
						Diameter	5–10 µm
						Bulk composition	Unknown

^a^ manufacturer data; ^b^ measured data.

## 4. Results and Discussion

### 4.1. Sedimentation

As mentioned earlier, ill-formulated MR fluids sediment due to the density mismatch between particles and the carrier fluid. In commercial MR fluids, stabilizers are added, which delay sedimentation but do not completely prevent it. Sedimentation can take minutes in MR fluids without additives, while it takes months in commercial ones. The settling of particles is an important issue because fast sedimentation deteriorates performance. There is a considerable industrial interest in monitoring sedimentation with a robust, online, and non-invasive technique.

**Figure 5:**
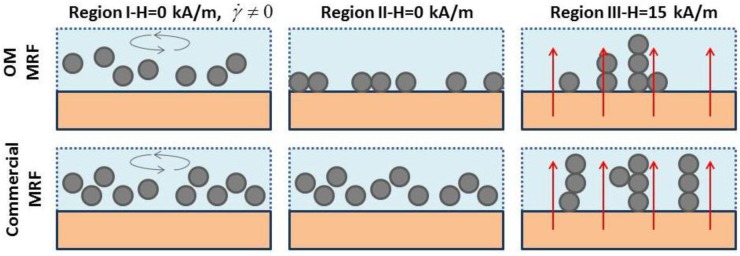
Scheme of possible structures formed in OM MRF and commercial MRF at the pre-shear stage-region I (**Left**), at rest -region II (**Middle**), and under the effect of a magnetic field-region III (**Right**).

**Figure 6:**
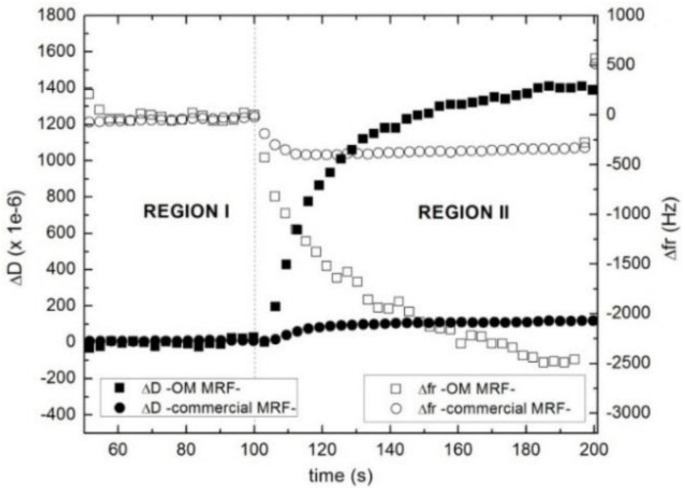
Dissipation (full dots) and resonance frequency shift (open dots) of OM MRF (squares) and commercial MRF (circles) as a function of time at 8 MHz. In region I (pre-shear stage) the MR fluids are subjected to a constant shear rate (100 s^−1^). In region II, the shear is turned off, letting the structure approach equilibrium.

A two-step protocol was employed to study sedimentation. In a first “pre-shear” stage (region I in Figure 5 and Figure 6) the sample was subjected to a high shear by rotating the upper plate at a 100 s^−1^ constant angular velocity. This homogenizes the suspension and erases all memory to the sample’s history. Subsequently, the rotation is stopped (region II in Figure 5 and Figure 6) and the system is allowed to evolve in the quiescent state.

Figure 5 shows a scheme of the possible structures formed in OM MRF and in the commercial MRF for the three different regions. When the system is at rest with no magnetic field applied (center), particles settle (OM MRF) or remain suspended (commercial MRF). When applying a magnetic field (right), one forms a chain-like structure with chains aligned to the field (vertical direction).

Figure 6 shows the changes in dissipation (Δ*D*) and the resonance frequency shifts (Δ*fr*) during the pre-shear stage (region I) and the rest stage (region II) of OM MRF (squares) and the commercial MRF (circles). While shear is applied (region I), Δ*f* and Δ*D* remain constant. The shear flow distributes particles evenly in the fluid volume, so that the system behaves like a fluid. The sensor sees a fluid with average properties that are constant in space and time.

When the shear flow is switched off (region II), Δ*fr* drops, while Δ*D* increases. This happens in both cases, but the changes are more pronounced for the sample OM MRF. When a non-stabilized suspension like the OM MRF is left alone without being stirred, it immediately sediments. This implies that an increasing number of particles make contact with the sensor surface, which increases the apparent viscosity and density of the fluid close to the surface. The negative shift of the resonance frequency shows that inertial effects are larger than elastic interactions. In principle, one expects elastic interactions to also be of influence at point contacts between the particles and the surface.

**Figure 7:**
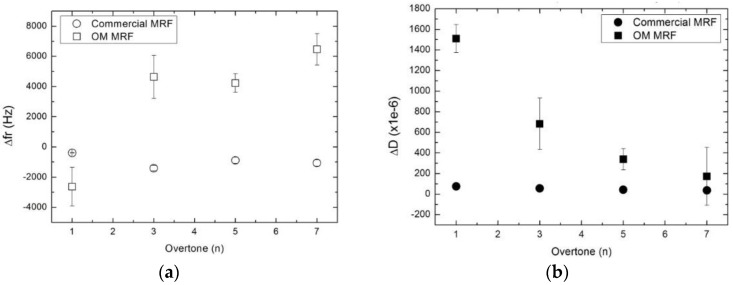
(**a**) Frequency shift (open) and (**b**) change in dissipation (solid) as function on the overtone at the end of the region II. Values shown are referenced to the end of the region I.

There is a second effect, which is only observed in the case of the commercial MRF. The thixotropic agents prevent the commercial suspension from settling. Rather, the cessation of shear leads to an increase in the viscosity as a result of the strong shear-thinning character of this material. This is the most plausible explanation for the changes observed in Δ*fr* and Δ*D* seen for this MR fluid. Note that this process is completed in about 10 s (circles in Figure 6), while the sedimentation proceeds all through stage II. This interpretation is corroborated by the overtone dependence of Δ*fr* and Δ*D* shown in Figure 7. For the OM MRF, Δ*fr* crosses from negative to positive between the fundamental frequency and the higher overtones, as is characteristic for the coupled resonance (Figure 1). At the same time, Δ*D* decreases with overtone order, which also agrees with this picture. For a more detailed justification of the last statement, see Ref. [3] and also references [24,25]. In contrast, Δ*fr* is negative at all overtone orders for the commercial MRF. Clearly, there is no coupled resonance in this case. Rather, the material behaves a semi-infinite viscoelastic medium. The particles in this fluid are suspended instead of being settled down.

### 4.2. Effects of Magnetic Fields

**Figure 8:**
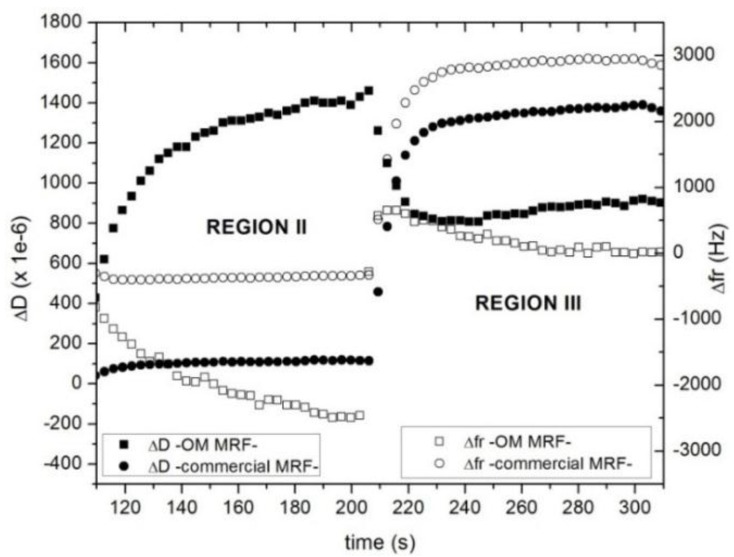
Dissipation (solid) and resonance frequency shift (open) of OM MRF 5% (squares) and commercial MRF 32% (circles) as function of time at 8 MHz. In the first step (region II) the shear rate is set to zero, letting the structure evolves to equilibrium after the pre-shear rate. In the second step (region III), a 15-kA/m magnetic field is applied perpendicularly to the rheometer plates.

As described in the previous section, the behavior at rest is very different from one MR fluid (OM MRF) to the other (commercial MRF). After the pre-shear stage (region I), the sample OM MRF sediments, while the commercial MRF does not (region II). These different starting points are key to understand the changes in dissipation and in the resonance frequency when the magnetic field is eventually applied. Figure 8 shows that the particles in OM MRF pass from being in contact to the QCM—with a rather loose interaction as they are just settling in region II—to be rearranged in a certain structure aligned with the external magnetic field in region III. Presumably, the increased amount of order also increases interaction between the particles and the surface. These changes result in an increase of the resonance frequency and in a decrease of dissipation (Figure 8). This result agrees with an increase of the elastic interaction contact which favors the resonance response.

However, in the case of the commercial MRF, there is a significant increase of both Δ*D* and Δ*fr* as the system changes from a non-contact situation (region II) to a strong interaction between the structure formed and the QCM surface (region III). This evolution implies an increase in rigidity (and, in consequence, an increase of resonant frequency), as well as an increase of dissipation. Δ*fr* is positive because the contacts increases the effective stiffness of the composite resonator. Chain formation avoids rotation of the particles in contact with the surface [3,12,24,26].

**Figure 9:**
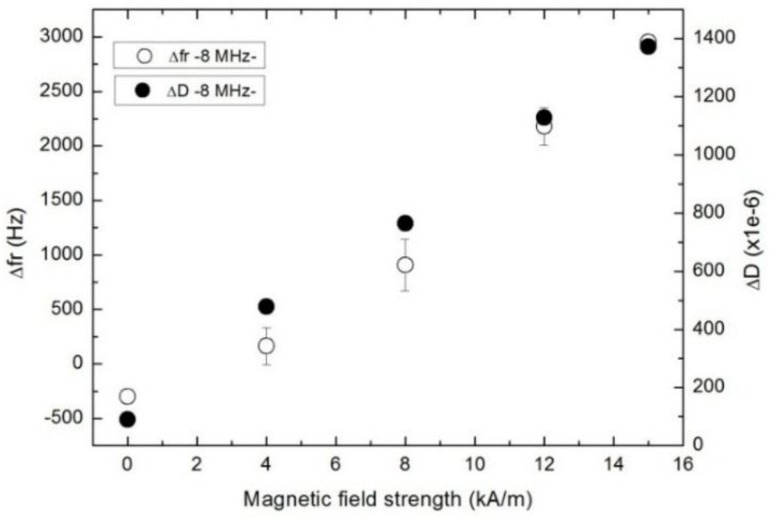
Change in dissipation (solid) and in resonance frequency (open) as a function of the magnetic field intensity applied to the commercial MRF at 8 MHz.

The effect of the magnetic field strength on the interaction between particles and the QCM surface and within the particles forming the structure was analyzed carrying out experiments at different magnetic field intensities using the commercial MRF at the fundamental frequency (Region III). The trend is clear and consistent: the higher the magnetic field strength, the higher the changes in dissipation and resonance frequency shifts (Figure 9). Note: The changes are referenced to the state in Region I, where particles are suspended in the fluid (as opposed to region II).

These results can be understood in the coupled-resonance frame. Generally speaking, magnetic fields cause stringing. Magnetic dipoles are oriented along the direction of the field and the dipole-dipole interactions force these aligned dipoles to form chains. For the particle volume fraction present in this MR fluid, a more complex structure is formed due to the interactions taking place transversely between chains. That provide for a stronger elastic (reactive) contribution to the forces acting at the surface. An increase of the magnetic field intensity rearranges the particles generating a more rigid structure. The structure is tightly attached to the sensor surface, limiting its movement.

By-and-large, the QCM does not see the bulk medium. In a liquid medium, the depth of penetration of the shear wave is below a micron. Particles transport stress more efficiently than a liquid, but the QCM still does not see the chains as a whole. However, QCM may indirectly sense particles further away from the surface, where the interaction is mediated by the particles in contact with the resonator. This hypothesis can explain the hindrance of movement and rotation as a factor for the increase in Δ*fr* with increasing magnetic field. Remember that two regions can be distinguished: the deformed region of the sphere (close to the contact) and far-field region with no or little deformation. The bulk of the sphere is at rest for large enough particles, while it moves for smaller ones. Importantly, the movement can be assumed to be mostly given by a rotation about the sphere center for larger particles immersed in liquid. An oscillatory rotation about the particle center allows the point of contact to follow the motion of the resonator surface, but at the same time, it avoids translation and pressure waves being launched into the liquid.

**Figure 10:**
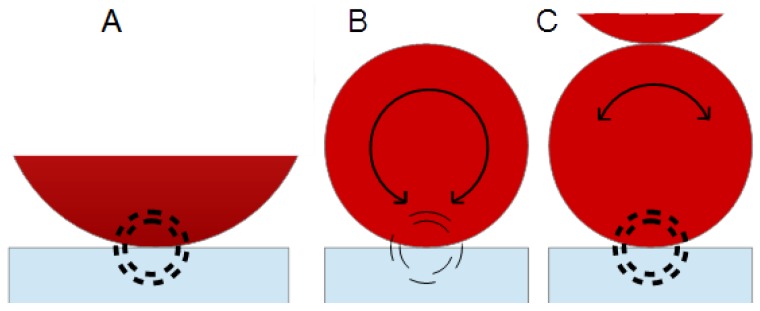
Scheme of particle-surface interaction. (**A**) A particle at rest; (**B**) particles which can rotate; and (**C**) a particle, the rotation of which is hindered by the presence of second particle touching it at the top (which is part of the same chain-like structure).

The inhibition of the rotation provides for a mechanism, by which the QCM can actually sense the second particle. The situation is sketched in Figure 10. Panel A depicts the elastic limit. A large sphere is at rest. The deformed zone is indicated with dashed circles. It constitutes a spring. Panel B shows a single particle, which is small enough to undergo some rotation. Due to the rotation, the bottom of the closest particle follows the resonator’s motion. The stiffness of the spring is the same as before, but the amount of shear deformation at the link is reduced. At the same time, the link is bent. In consequence, the force exerted onto the resonator surface is reduced, which also reduces the (positive) frequency shift. Panel C shows the same particle, but now in contact with a second particle at the top. The two particles are part of a string, generated by the magnetic field. Due to the increase of the stiffness of the structure, the second sphere impedes the rotation of the first, thereby increasing the force exerted by the link and—in consequence—the frequency shift.

As the magnetic field increases, the dissipation is found to increase, as well. As the spheres form chains, the rigidity of the entire structure is increased. This facilitates the transport of stress away from the resonator surface and enlarges the amount of dissipated energy.

In the discussion above, plausible assumptions were made on the contact mechanics which, in conjunction with the experimental values of Δ*fr* and Δ*D*, were used to infer the state of the sample. The line of the argument might be turned around: if the focus is on contact mechanics rather than MR fluids, well-characterized and well-understood MR fluids can be a useful tool to study contact mechanics, rather than making assumptions about it. An MR fluid subjected to magnetic fields of variable strength amounts to a convenient way of tuning the interactions between the particles and the surface.

### 4.3. Finite Element Modeling

We have used finite element modeling (FEM) to simulate the resonant frequency shifts of the two fluids when an external magnetic field is applied. We have modeled two situations for the OM MRF fluid. Firstly, the case where the settling of particles occurs and, secondly, the case in which a magnetic field is applied. The methodology of this calculation is described below.

**Figure 11:**
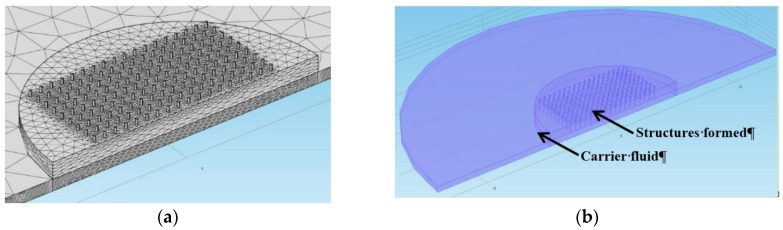
Images taken from the COMSOL model interface (**a**) mesh (centered on the center of the electrode); and (**b**) geometry.

A 3D FEM model (*COMSOL Multiphysics 4.4*) has been performed to evaluate the influence of structure-surface interaction on the resonant frequency of an AT-cut quartz crystal (described in Section 3.1), which vibrates in the fundamental thickness-shear mode at 8 MHz (thickness, 0.208 mm), and takes into account both the crystal anisotropy and the piezoelectric effect. As a first step, the matrices of natural quartz are set up, and afterwards the coordinate system is rotated about the “y” axis by 35.25° to obtain the AT-cut. The number of meshing elements in each direction was optimized to get correct results, while taking into account existing resources. A total of 125,954 elements were used (Figure 11a). Symmetry about the “y” axis was applied to simplify the model (Figure 11b).

The carrier fluid was modeled as a highly viscous oil (shear loss modulus *G’’ =* 25 MPa at 8 MHz and density ρ = 1050 Kg/m^3^). Structures formed with particle settling were modeled with G’ = 5 MPa, G’’= 5 MPa and ρ = 3000 Kg/m^3^. Structures formed under the effect of a magnetic field were modeled increasing the shear storage module G’ = 50 MPa maintaining G’’ = 5 MPa and ρ = 3000 Kg/m^3^. In both cases the modeled structures have a diameter of 50 μm, and the distance between them is 200 μm. Both situations have been modeled by changing the value of the parameter G′ following a simple series connection mechanical model between the metallic particles and the carrier fluid. In the case of settling, the particles form a non-consolidated structure placed over the sensor and, in the second case, the same particles form a more rigid structure due to the magnetic field. A strong assumption is made maintaining the same structure for the two cases, changing only the material properties of the constituent material. This structure is compatible with those observed in the OM MRF fluid by microscopy when applying a magnetic field perpendicular to the resonator plane. Most relevant results are contained in Table 2.

**Table 2:** Resonant frequency shifts of the quartz resonator calculated for settling and external magnetic field conditions (Note: the changes are referenced to the case of carrier fluid placed on the resonator).

Description	Δfr FEM Results
Magnetic field applied	950 Hz
Sedimentation	−1000 Hz

According to FEM (see Table 2), a negative shift of −1000 Hz is obtained when sedimentation occurs, whereas a positive resonant frequency shift of 950 Hz is obtained when magnetic field is applied. In Figure 6, it was shown an experimental negative resonant frequency shift of 2500 Hz, while in Figure 8, it was shown a positive shift of 650 Hz when the external magnetic field was applied. After the time the field is applied, the frequency decreases slowly because the structure evolves because the shaking of the resonator introduces some structure reorganization. The model describes well the sign of the frequency shift but is not accurate to predict the experimental frequency shift obtained. This is not surprising because of the simplifications of the model and parameter estimations; in experimental cases, there are a huge number of contacts which non-constant rigidity properties. Nevertheless, these numeric results supports the validity of the micro-contact theoretical assumptions made to explain the resonator behavior when measuring MR fluids.

## 5. Conclusions

TSM resonators are a robust, widely applicable, and non-destructive tool to study MR fluids. Although they are surface specific, bulk properties can be inferred from the data obtained by these resonators. In particular, effects of sedimentation, as well as changes of viscosity caused by external fields, are readily observed because both effects alter the interaction between the particles and the resonator surface.

This is the first time that MR fluids are studied with this technique. Heavy microparticles which can be rearranged by magnetic fields provide for a tunable system, which facilitates the study of the coupled resonance. It was shown that both regimes can be distinguished with the QCM as a function of frequency when particles sediment towards the sensor, and consequently the number of microcontacts is increased. For instance, in the case of OM MRF, at the fundamental frequency (8 MHz), we have measured a decrease of the resonance frequency of 2500 Hz. At higher overtones, however, the resonance frequency is increased up to 6500 KHz (seventh overtone), as the system is in the elastic regime according to the coupled resonance model.

Being a suspension of ferromagnetic microparticles subjected to an external magnetic field, interactions among particles and interactions between these particles and the resonator surface increase when the magnetic field is also increased. This leads to positive shifts of 650 Hz at the fundamental mode for FMR_OM. These results are in accordance with an increase of the stiffness of the contacts. The inhibition of the particle rotation due to the formation of the rigid chain structure also contributes to the increase of the resonance frequency and dissipation. For instance, in the case of commercial MRF, this dependence with increasing magnetic field was showed.

A FEM model was used to simulate the effect of particle contacts on the resonator response based on the stiffness changes predicted by the theoretical model for the case of the OM MRF. Both cases of free settling of particles and particle alignment under the action of the magnetic field were modeled. Frequency shift trends obtained are in agreement with the experimental results shown in this work. The exact frequency shifts obtained by experimental measurement and by our model are not exactly the same due to the fact that the numerous contacts vary in their properties.
